# Efficacy of 0.12% Chlorhexidine and *Salvadora persica*-based Mouthwash in Reducing Oral Candida Carriage and Periodontal Inflammation in Cigarette Smokers and Non-smokers after Non-surgical Periodontal Therapy

**DOI:** 10.3290/j.ohpd.b4169713

**Published:** 2023-06-20

**Authors:** Amani M. Basudan, Abeer S. Al-Zawawi, Darshan Devang Divakar, Marwa Y. Shaheen, Hajer A. Aldulaijan

**Affiliations:** a Assistant Professor, Department of Periodontics and Community Dentistry, College of Dentistry, King Saud University, Riyadh, Saudi Arabia. Designed the study, supervised the research project, wrote the manuscript, read and approved the manuscript prior to submission.; b Assistant Professor, Department of Oral Medicine and Radiology, Sharavathi Dental College and Hospital, Shivamogga, Karnataka, India; Department of Oral Medicine and Radiology, Faculty of Dentistry, Levy Mwanawasa Medical University (LMMU), Ministry of Health, Lusaka, Zambia. Clinical and laboratory investigations, mechanical debridement, administered the questionnaire, statistical analysis, read and approved the manuscript prior to submission.; c Assistant Professor, Department of Periodontics and Community Dentistry, College of Dentistry, King Saud University, Riyadh, Saudi Arabia. Wrote the manuscript.; d Assistant Professor, Department of Periodontics and Community Dentistry, College of Dentistry, King Saud University, Riyadh, Saudi Arabia. Wrote the manuscript, read and revised the manuscript prior to submission.

**Keywords:** chlorhexidine, mouthwash, non-surgical periodontal treatment, periodontal inflammation, Salvadora persica, smoking

## Abstract

**Purpose:**

The present study assessed the efficacy of 0.12% chlorhexidine (CHX) and *Salvadora persica*-based mouthwashes (SPM) in reducing oral *Candida* carriage (OCC) and periodontal inflammation in cigarette smokers and non-smokers after non-surgical periodontal treatment (NSPT).

**Materials and Methods:**

Self-reported cigarette smokers and non-smokers with periodontal inflammation as well as non-smokers with a healthy periodontal status were included. NSPT was performed in all participants. Based on the type of mouthwash, participants were randomly divided into three groups as follows: group 1: CHX; group 2: SPM; and group 3: distilled water (ddH_2_O) with mint flavour (control group). Clinical attachment loss (CAL), plaque index (PI), gingival index (GI), probing depth (PD), and marginal bone loss (MBL) were measured. Clinical periodontal parameters were re-assessed at a 6-week follow-up. Oral yeast samples were collected and identified using a concentrated oral-rinse culture technique and PCR, respectively. Clinical and laboratory-based investigations were done at baseline and after six weeks. Statistical significance was set at p < 0.05.

**Results:**

At baseline, PI, MBL, PD and CAL were comparable in all participants. None of the patients had periodontitis at baseline. Post-operatively, CHX and SPM were more effective in reducing PI (p < 0.01), GI (p < 0.01) and PD (p < 0.01) in non-smokers than in the control group. The OCC was statistically significantly higher among smokers compared with non-smokers at baseline. At the 6-month follow-up, CHX was more effective than SPM in reducing OCC in non-smokers (p < 0.01). At the 6-week follow-up, there was no difference in OCC among cigarette smokers regardless of the type of mouthwash prescribed postoperatively.

**Conclusion:**

In cigarette smokers and non-smokers, CHX and SPM are effective in reducing periodontal soft-tissue inflammation after NSPT. Post-operative use of CHX is more effective than SPM in reducing OCC.

A compromised oral hygiene status is the most common risk factor for periodontal inflammation.^3,38^ Another local factor that has been linked with the aetiology and progression of oral diseases, e.g. periodontal disease, is habitual use of combustible tobacco products such as cigarettes.^20^ Studies^12,20,34,46^ have shown that cigarette smokers are more susceptible to periodontal diseases compared with non-smokers. Moreover, from a mycological point of view, poor oral hygiene maintenance and habitual smoking are associated with an increase in the counts and colonisation of oral yeast species (predominantly *Candida albicans*), which otherwise is part of the commensal oral flora.^22,30,32,45^ It is speculated that an increase in the counts of oral yeasts may potentially predispose vulnerable patient populations to oral diseases such as periodontal disease and candidiasis.

Non-surgical periodontal treatment (NSPT) involves mechanical debridement of teeth and root surfaces and periodontal pockets; and is the most common therapeutic protocol used for treating periodontal inflammatory conditions.^3^ As a prophylactic measure, oral rinses or mouthwashes are often used by individuals for routine oral hygiene maintenance. Moreover, mouthwashes such as 0.12% chlorhexidine (CHX) are also prescribed by dental professionals following NSPT. Although usage of CHX is a safe and effective prophylactic pharmaceutical agent for the management of oral inflammatory conditions such as gingivitis and periodontitis, it may sometimes trigger unpleasant symptoms such as a burning sensation in mouth, tooth staining, and mucositis in some patients.^25,36,40^ In a recent randomised control trial (RCT), Al-Zawawi et al^3^ showed that mouthwashes based on natural products such as essential oils are a suitable replacement for CHX in patients with CHX allergy. One justification for this is that essential oil extracts exhibit anti-inflammatory, anti-nociceptive and anti-microbial properties.^18,24,44^ *Salvadora persica* (*S. persica*), commonly known as miswak in the Middle-Eastern world, is a medicinal herb that exhibits anti-inflammatory, antibacterial, and antioxidant effects.^9,29,35^ Clinical studies^2,23^ have shown that *S. persica*-based mouthwash (SPM) improves periodontal health and facilitates plaque control. However, results from a recent RCT^2^ reported that the anti-inflammatory efficacy of SPM is lower than that of CHX. From a mycological perspective, results from in-vitro studies^5,35^ have shown that *S. persica* extracts are natural inhibitors of *Candida* growth. Clinical studies^10,43^ have also shown that *S. persica* extracts contribute to reduction in plaque and gingival indices (PI and GI, respectively). A meticulous review of pertinent indexed literature showed that no studies exist which have compared the anti-inflammatory and anti-fungal efficacy of CHX and SPM in cigarette smokers and non-smokers with periodontal inflammation.

The present study assessed the efficacy of 0.12% CHX and a SPM in reducing oral *Candida* carriage (OCC) and periodontal inflammation in cigarette smokers and non-smokers after NSPT. The null hypothesis is that there is no difference in the anti-inflammatory and anti-fungal efficacy of CHX and SPM in cigarette smokers and non-smokers with periodontal inflammation.

## Materials and Methods

### Ethics Approval and Consent to Participate

The 2013-revised Guidelines of the Declaration of Helsinki involving human subjects were followed. Individuals were provided written information about the objectives and methodology of the present investigation. All subjects were asked to read and sign a written informed consent form. All individuals were also aware that refusal to participate or withdrawal at any stage did not bear consequences. This RCT was registered and approved by the ethics research committee of the Sharavathi Dental College and Hospital, Shivamogga, Karnataka, India (approval # SDC/SMG/2022148). Irrespective of their decision to participate in the present study, all individuals were educated about the detrimental effects of smoking on overall health, and the importance of routine oral hygiene for a healthy lifestyle.

### Eligibility Protocol

Self-reported cigarette smokers and non-smokers with periodontal inflammation as well as non-smokers with a healthy periodontal status were included. Cigarette smokers were defined as individuals who had smoked at least one cigarette daily for at least the past twelve months.^4^ Participants that reported to have never used any type of nicotinic/tobacco product were classified as ‘non-smokers’.^4^ Periodontal inflammation was defined using the following parameters in at least 30% sites: (1) presence of dental plaque and gingival bleeding upon gentle probing; (2) probing depth (PD) of at least 3 mm; (3) clinical attachment loss (CAL) of at least 1 mm.^11^ A healthy periodontal status was defined as no CAL, PD < 3 mm, and gingival bleeding at ≤ 10% sites.^13^ Dual-smokers and individuals using other forms of combustible and smokeless tobacco-product users (such as waterpipe, pipe, cigar and snuff, respectively) were not included. Moreover, patients with self-reported systemic illnesses, such as respiratory, metabolic, or cardiovascular diseases, were excluded. Habitual alcohol usage, refusal to sign the consent form, and pregnancy/lactation were also exclusion criteria. Individuals who reported to have consumed medications (such as bisphosphonates, steroids, probiotics, antibiotics, and non-steroidal anti-inflammatory drugs) were not included. From a dental perspective, third molars, supernumerary teeth and remaining root remnants were not evaluated and were considered missing.

### Study Design and Groups

A single-blinded parallel-arm design was adopted in the current investigation. Based upon baseline periodontal status, patients were classified as: (1) cigarette smokers with periodontal inflammation; (2) non-smokers with periodontal inflammation; (3) non-smokers without periodontal inflammation. Individuals in each group were further sub-classified into three groups depending on the type of mouthwash prescribed: (a) non-alcoholic 0.12% CHX group; (b) non-alcoholic SPM (Himalaya Drug Company, HiOra; Bengaluru, India) group; (c) control group: distilled water with mint flavour.

### Allocation Concealment, Randomisation, and Blinding

Allocation concealment was carried out by one author (DDD) using an independent computer-generated randomisation sequence process that was only accessible to the study supervisor. The mouthwashes were placed in an opaque plastic bottle with a lid, possessing standardised dimensions, colour, and shape. The mouthwashes were given to study participants by an investigator blinded to the contents.

### Study Location and Patient Demographics

This study was done at the outpatient department of the College of Dentistry, Sharavathi Dental College, and Hospital, Shivamogga, Karnataka, India, between August 2021 and March 2022. Patient demographics (sex, age, brushing and flossing habits, systemic health status, most recent visit to an oral healthcare provider, and smoking habit) were recorded using a questionnaire which was presented to all participants by one investigator.

### Non-surgical Periodontal Treatment

An ultrasonic scaler and sterile curettes (Hu-Friedy; Chicago, IL, USA) were used to performed NSPT without administration of local anesthesia by one trained investigator. These procedures were performed after assessment of baseline clinical parameters.

### Clinical and Radiographic Parameters

The PI^42^ and GI^28^ were measured at 4 sites per tooth: midbuccal, midpalatal/lingual, mesial and distal. CAL^7^ and PD^8,20^ were assessed in millimeters (mm) on 3 buccal (mesio-, mid-, and disto-) and 3 lingual/palatal (mesio-, mid-, and disto-) surfaces using a graded probe (UNC-15, Hu-Friedy). The number of missing teeth (MT) was also recorded. Clinical periodontal parameters were assessed at baseline (immediately before NSPT) and at the 6-week follow-up by a calibrated investigator (Kappa score 0.88). At baseline (immediately before NSPT), digital bitewing radiographs were taken for all teeth, and marginal bone loss (MBL) was measured to the nearest mm as the perpendicular distance from the cementoenamel junction to the alveolar crest.^20^

### Collection of Oral Yeast Samples and Determination of Oral *Candida* Carriage

Oral yeast samples were collected at baseline and at the 6-week follow-up by a trained investigator using a protocol described by Reichart et al.^37^. According to this protocol, the palate and dorsum of the tongue were swabbed with a sterile swab (Biomerieux; Montalieu-Vercieu, France), after which the swab was inserted into the alginate transport medium within the tube and immediately cultured at 37°C for 7 days in an aerobic environment on Sabouraud’s dextrose agar (Becton Dickinson; Sparks, MD, USA).^37^ The culture plates were inspected daily basis for yeast growth. *Candida* species were determined using polymerase chain reaction (PCR) and DNA sequencing. In summary, yeast cells were suspended in PCR-grade water (200 µl) for DNA isolation. A DNA preparation robot (MagNA pure, Roche Diagnostics; Mannheim, Germany) was used to attain genomic DNA. For DNA sequencing, PCR analysis was first performed by amplifying a region (approximately 500 bp) of the 18S-ribosomal ribonucleic acid gene using DNA polymerase and universal primers. Nucleotides were removed using a PCR purification kit (250 QIAquick Qiagen; Hilden, Germany), and DNA was sequenced using capillary electrophoresis (ABI 310 Genetic Analyzer, Applied Biosystems; Foster City, CA, USA). To minimise the possibility of error during sequencing, strands of PCR-amplified DNA fragments were sequenced. For yeast identification, DNA sequences were assessed using the BLAST-DNA database.

### Statistical Analyses and Sample Size Estimation

Quantitative assessment was done by a statistician who was blinded to the study groups. Data normality was determined using the Shapiro-Wilk test, and group comparisons were made using the one-way ANOVA and Bonferroni post-hoc adjustment tests. p-values less than 0.05 were considered statistically significant. A software programme (Statistical Solutions-nQuery Advisor-6; Saugas, MA, USA) was used for sample size estimation using data from a pilot study. It was estimated that inclusion of at least 28 patients per group would be needed to attain a study power of 85% with an alpha of 5% to detect a 2-mm difference in PD and 1-mm difference in CAL, considering a mean standard deviation (SD) of 0.5 mm.

## Results

### Recruitment of Study Participants

**Fig 1:**
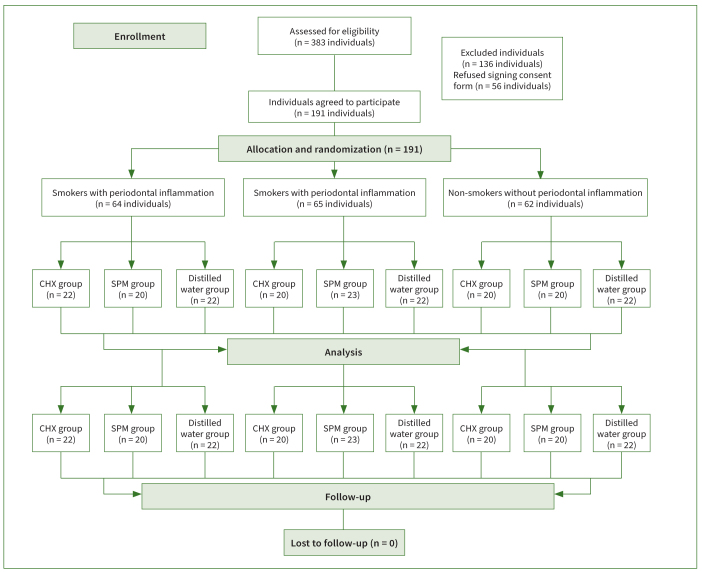
The study population.

Three hundred eighty-three (383) individuals were initially invited and screened for eligibility. One hundred thirty-six (136) individuals did not meet the eligibility criteria and were therefore excluded. Of the remaining 247 individuals, 56 individuals refused to sign the written informed-consent form. Collectively, 191 individuals (115 males and 76 females) agreed to participate in the present study and signed the written informed-consent form. Depending on their smoking and periodontal status, these individuals (n = 191) were divided into three groups: cigarette smokers with periodontal inflammation (n = 64), non-smokers with periodontal inflammation (n = 65), and non-smokers with a healthy periodontal status (n = 62). Among cigarette smokers, CHX, SPM and ddH_2_O were prescribed to 22, 20, and 22 individuals, respectively. Among non-smokers with periodontal inflammation CHX, SPM, and ddH_2_O were prescribed to 20, 23, and 22 participants, respectively. In non-smokers with a healthy periodontal status, CHX, SPM, and ddH_2_O were prescribed to 20, 20, and 22 participants, respectively (Fig 1).

### Demographics of the Study Groups

There was no statistically significant difference in the mean ages of individuals in all groups. On average, cigarette smokers were smoking 15 cigarettes daily for 21 years (15.7 pack years). There was no statistically significant difference in the smoking history (pack-years) among patients that received CHX, SPM, or hhH_2_O following NSPT. Toothbrushing twice daily was more often reported by non-smokers without periodontal inflammation in comparison with non-smokers and cigarette smokers with periodontal inflammation. Flossing of interproximal spaces and visiting a dentist or dental hygienist within the past 6 months was reported by none of the non-smokers and cigarette smokers with periodontal inflammation. Interdental flossing once daily was reported by at least 80% non-smokers without periodontal inflammation, and at least 70% of these individuals reported to have visited an oral healthcare provider within the past 12 months (Table 1).

**Table 1:** Demographic information

Demographic parameters	Cigarette smokers with periodontal inflammation				Non-smokers with periodontal inflammation				Non-smokers without periodontal inflammation			
	All patients	CHX group	SPW group	ddH_2_O group	All patients	CHX group	SPW group	ddH_2_O group	All patients	CHX group	SPW group	ddH_2_O group
Participants (n)	64	22	20	22	65	20	23	22	62	20	20	22
Male:Female ratio	36:28	12:9	12 :7	12:12	40:25	12:9	14:6	14:10	39:23	15:8	12 :8	12:7
Mean age in years	45.1± 7.3 years	48.4± 5.5 years	42.6± 6.4 years	44.6± 4.8 years	42.8± 3.6 years	44.8± 4.1 years	43.4± 2.5 years	40.5± 2.4 years	43.7± 4.4 years	45.6± 5.4 years	44.3± 4.7 years	41.2± 2.3 years
Smoking, pack-years	15.7	12.2	14.6	16.4	NA	NA	NA	NA	NA	NA	NA	NA
Toothbrushing 2x daily (n) (%)	14 (21.9%)	5 (22.7%)	4 (20%)	5 (22.7%)	20 (30.8%)	6 (30%)	8 (34.8%)	6 (27.3%)	57 (91.9%)	18 (90%)	20 (100%)	19 (86.4%)
Daily flossing 1x daily (n) (%)	—	—	—	—	—	—	—	—	50 (80.6%)	16 (80%)	18 (90%)	16 (72.7%)
Dental visit (within 12 months	—	—	—	—	—	—	—	—	48 (77.4%)	15 (75%)	14 (70%)	19 (86.4%)

—: no individuals; NA: not applicable.

### Clinical and Radiographic Periodontal Parameters at Baseline

At baseline, PI (p < 0.01), PD (p < 0.01), and MBL (p < 0.01) were statistically significantly higher among cigarette smokers and non-smokers with periodontal inflammation compared with non-smokers without periodontal inflammation. The GI (p < 0.01) was statistically significantly higher in non-smokers with periodontal inflammation compared with cigarette smokers with periodontal inflammation and non-smokers without periodontal inflammation. There was no statistically significant difference in PI, PD, CAL, and MBL among cigarette smokers and non-smokers with periodontal inflammation. There was no statistically significant difference in GI in cigarette smokers with periodontal inflammation and non-smokers without periodontal inflammation. There was no statistically significant difference in the numbers of MT in all groups (Table 2).

**Table 2:** Periodontal status at baseline

Periodontal parameters	Cigarette smokers with periodontal inflammation				Non-smokers with periodontal inflammation				Non-smokers without periodontal inflammation			
	All patients	CHX group	SPW group	ddH_2_O group	All patients	CHX group	SPW group	ddH_2_O grou	All patients	CHX group	SPW group	ddH_2_O group
Plaque index	0.78±0.05*	0.7±0.07*	0.8±0.1*	0.8±0.06*	0.7±0.1	0.6±0.07	0.7±0.08	0.8±0.1	0.35±0.05	0.3±0.07	0.2±0.005	0.3±0.04
Gingival index	0.4±0.1†	0.4±0.05†	0.3±0.02†	0.3±0.06†	0.77±0.1*	0.8±0.1*	0.7±0.04*	0.8±0.04*	0.3±0.003	0.21±0.02	0.36±0.04	0.2±0.02
Probing depth	4.4± 0.1* mm	4.6± 0.07 mm*	4.3± 0.04 mm*	4.5± 0.1 mm*	4.5± 0.2 mm	4.6± 0.05 mm	4.3± 0.09 mm	4.4± 0.08 mm	1.5± 0.05 mm	1.1± 0.1 mm	1.2± 0.07 mm	1.1± 0.08 mm
Clinical attachment loss	1.3± 0.05 mm	1.2± 0.05 mm	1.4± 0.06 mm	1.2± 0.02 mm	0.8± 0.03 mm	0.7± 0.08 mm	1.2± 0.05 mm	1.08± 0.04 mm	None	None	None	None
Marginal bone loss (mesial)	1.6± 0.2 mm*	1.8± 0.1 mm*	1.8± 0.07 mm*	1.7± 0.06 mm*	1.6± 0.1 mm*	1.7± 0.07 mm	1.4± 0.05 mm	1.7± 0.1 mm	0.4± 0.1 mm	0.3± 0.07 mm	0.5± 0.04 mm	0.2± 0.02 mm
Marginal bone loss (distal)	1.5± 0.05 mm*	1.6± 0.06 mm*	1.5± 0.03 mm*	1.4± 0.02 mm*	1.6± 0.2 mm	1.7± 0.05 mm	1.4± 0.04 mm	1.5± 0.06 mm	0.2± 0.05 mm	0.1± 0.005 mm	0.4± 0.02 mm	0.2± 0.1 mm
Missing teeth	3.2± 0.2 teeth	3.6± 0.1 teeth	2.7± 0.5 teeth	3.1± 0.08 teeth	4.1± 0.2 teeth	3.8± 0.06 teeth	3.5± 0.1 teeth	4.5± 0.04 teeth	1.2± 0.1 teeth	None	1.4± 0.1 teeth	1.6± 0.1 teeth

*Compared with non-smokers without periodontal inflammation (p < 0.01). †Compared with non-smokers with periodontal inflammation (p < 0.01).

### Clinical Periodontal Parameters at Follow-up

**Fig 2:**
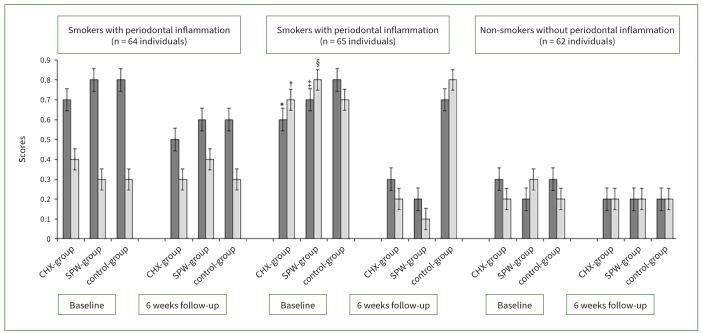
Plaque (dark grey bars) and gingival (light grey bars) indices in the study groups at baseline and 6-week follow-up. *Compared with CHX group at 6-week follow-up among non-smokers with periodontal inflammation (p < 0.01). †Compared with CHX group at 6-week follow-up among non-smokers with periodontal inflammation (p < 0.01). ‡Compared with SPM group at 6-week follow-up among non-smokers with periodontal inflammation (p < 0.01). §Compared with SPM group at 6-week follow-up among non-smokers with periodontal inflammation (p < 0.01).

**Fig 3:**
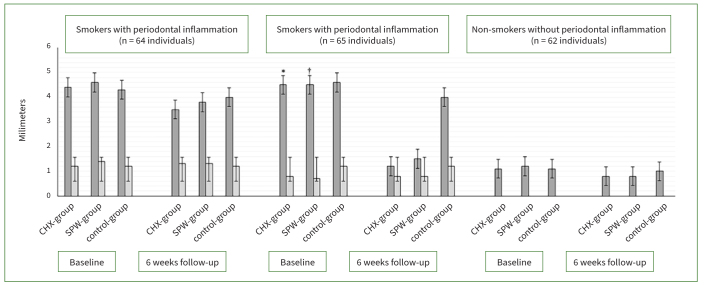
Probing depth (dark grey bars) and clinical attachment loss (light grey bars) in the study groups at baseline and 6-week follow-up. *Compared with CHX group at 6-week follow-up among non-smokers with periodontal inflammation (p < 0.01). †Compared with SPM group at 6-week follow-up among non-smokers with periodontal inflammation (p < 0.01).

Irrespective of the type of mouthwash prescribed, there was no statistically significant difference in PI, GI, PD and CAL among cigarette smokers with periodontal inflammation and non-smokers without periodontal inflammation compared with their respective baseline scores. There was a statistically significant reduction in PI (p < 0.01), GI (p < 0.01), and PD (p < 0.01) among non-smokers with periodontal inflammation that were prescribed CHX and SPM vs individuals who were prescribed ddH_2_O after NSPT. There was no statistically significant difference in PI, GI, PD and CAL among non-smokers with periodontal inflammation that were prescribed CHX and SPM. In all participants, there was no statistically significant difference in periodontal parameters in the control group (ddH_2_O group) when baseline values were compared with 6-week follow-up results (Figs 2 and 3).

### Oral Yeast Carriage at Baseline

At baseline, oral yeast species were identified in all cigarette smokers with periodontal inflammation and 92.3% and 46.8% non-smokers with and without periodontal inflammation, respectively. The most common oral yeast species identified in the study population was *C. albicans*, which was isolated from 89.1% and 58.5% cigarette smokers and non-smokers with periodontal inflammation, respectively. Among individuals without periodontal inflammation, *C. albicans* was isolated from 35.5% individuals at baseline. *C. albicans* was more often isolated from the oral cavity of cigarette smokers and non-smokers with periodontal inflammation compared with non-smokers without periodontal inflammation. The second most common yeast species identified in the study groups was *C. tropicalis*. Among cigarette smokers and non-smokers with periodontal inflammation, *C. parapsilosis* was identified in one (1.6%) and 6 (9.2%) individuals, respectively (Table 3).

**Table 3:** Oral yeast carriage at baseline

Oral yeast species	Baseline			6-week follow-up		
	Cigarette smokers with periodontal inflammation (n = 64)	Non-smokers with periodontal inflammation (n = 65)	Non-smokers withoutperiodontal inflammation (n = 62)	Cigarette smokers with periodontal inflammation (n = 64)	Non-smokers with periodontal inflammation (n = 65)	Non-smokers without periodontal inflammation (n = 62)
*Candida albicans* (n) (%)	57 (89.1%)	38 (58.5%)	22 (35.5%)	46 (71.9%)	21 (32.3%)	20 (32.3%)
*Candida tropicalis* (n) (%)	6 (9.4%)	16 (24.6%)	7 (11.3%)	14 (21.9%)	5 (7.7%)	6 (9.7%)
*Candida parapsilosis* (n) (%)	1 (1.6%)	6 (9.2%)	None	4 (6.3%)	None	None
No yeast growth	None	5 (7.7%)	33 (53.2%)	None	39 (60%)	36 (58%)

**Fig 4:**
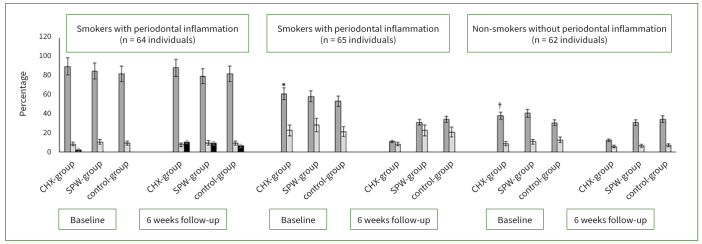
Oral *Candida albicans* (dark grey bars), *Candida tropicalis* (light grey bars) and *Candida parapsilosis* (black bars) carriage among participants who were post-operatively prescribed CHX, SPM and ddH_2_O. *Compared with CHX group at 6-week follow-up (p < 0.01). †Compared with CHX group at 6-week follow-up (p < 0.01).

At the 6-week follow-up, OCC was 2.2 times higher in cigarette smokers vs non-smokers with and without periodontal inflammation. There was no statistically significant difference in OCC in cigarette smokers who were prescribed CHX, SPM or ddH_2_O following NSPT. Among non-smokers with periodontal inflammation, oral *C. albicans* (p < 0.01) and *C. tropicalis* (p < 0.01) carriage was statistically significantly higher in the SPM and ddH_2_O groups compared with individuals who were prescribed CHX. Among non-smokers with a healthy periodontal status, there was a statistically significant reduction in *C. albicans* carriage among patients that were prescribed CHX (p < 0.01) compared with those who were prescribed SPM and ddH_2_O after NSPT. At the 6-week follow-up, there was no statistically significant difference in OCC among non-smokers who received SPM and ddH_2_O after NSPT (Fig 4).

### Correlation Between Age, Gender, Periodontal Parameters, Smoking and Oral Yeast Carriage

There was no statistically significant correlation between OCC and age, gender, smoking history (pack-years), and clinical periodontal parameters (data not shown).

## Discussion

It is important to clarify that in the present investigation, there were no stringent criteria to exclusively include cigarette smokers with periodontal inflammation. It would have been interesting to identify cigarette smokers without periodontal diseases and evaluate them from a periodontal and mycological perspective; however, based upon the periodontal investigations performed in the current investigation, none of the cigarette smokers were devoid of periodontal inflammation. This finding confirms previously known evidence that cigarette smoking increases the risk of periodontal diseases in susceptible patient populations. Habitual cigarette smoking has been shown to increase the expression of inflammatory proteins, such as advanced glycation end-products and pro-inflammatory cytokines in oral fluids (e.g. whole saliva).^17,31^ Based on this explanation, it was anticipated that periodontal parameters would be poorer among cigarette smokers than non-smokers with periodontal inflammation at baseline. However, the present results showed no statistically significant difference in PI, GI, PD, MT, CAL and MBL. One clarification for this is that cigarette smokers that agreed to participate in the present study were ‘light smokers’,^27^ as they had a smoking history of approximately 16 pack-years. This could also explain why none of the cigarette smokers had periodontitis. It is therefore anticipated that periodontal inflammation and severity of periodontitis is worse in heavy smokers with a smoking history of over 40 pack-years. Moreover, it is known that severity of periodontal disease increases with advancing age.^15,20,26^ According to Javed et al,^20^ periodontal inflammation is worse in individuals over 60 years old vs individuals 45 to 49 years of age. In the present clinical investigation, all participants were relatively young (mid-40s). Furthermore, nicotine reduces gingival blood flow, thereby masking the clinical signs of periodontal disease among smokers.^14^ This could serve as an explanation for the comparable periodontal parameters among smokers and non-smokers at baseline. Overall, from a clinical periodontal perspective, CHX as well as SPM are effective in reducing periodontal inflammation in susceptible patients. The results of Al-Zoman et al^6^ showed that CHX and SPM are effectivex in reducing gingival bleeding, PD and formation of dental plaque. Herbal mouthwashes are a potential replacement for patients with CHX allergy, as reported in a recent clinical investigation.^3^

With reference to OCC, the present results showed no statistically significant difference among cigarette smokers regardless of the type of mouthwash prescribed postoperatively. This outcome contradicts previous experimental investigations,^5,33,35,43^ which demonstrated that extracts from herbs including *S. persica* are natural anti-antifungal agents. It is well known that tobacco smoking is a risk factor for increased OCC.^1,45^ Moreover, nicotine (a major and addictive constituent of tobacco) increases biofilm thickness as well as yeast adherence.^16^ It is therefore likely that the cigarette smokers included in the current investigation continued to smoke for at least until the 6-week follow-up period. This offers an explanation of the present results, which showed no statistically significant reduction in OCC among cigarette smokers from baseline to follow-up. An interesting finding was that among non-smokers, the anti-fungal efficacy of CHX was higher than that of SPM. In summary, the 6-week follow-up results shown in Fig 4 showed a statistically significant reduction in OCC among non-smokers who were prescribed CHX in contrast to ddH_2_O and SPM. Our clinical results are in accordance with a previous in-vitro experiment by Siddeeqh et al,^41^ who concluded that the antimicrobial effectiveness of SPM is ‘less’ than that of CHX. According to those authors, alcoholic extracts of *S. persica* are more effective antimicrobial agents than aqueous *S. persica* extracts. Jose et al^21^ also reported that alcoholic extracts of medicinal plants demonstrate a stronger inhibition of pathogenic bacteria than do aqueous extracts. Since alcohol is a better solvent than water for extracting bioactive herbal compounds, it is likely that alcohol-based herbal mouthwashes exert a stronger antibacterial and anti-fungal effect than do non-alcoholic herbal mouthwashes. It is worth mentioning that the SPM used in the present study was non-alcoholic; this factor seems to have compromised the anti-*Candida* efficacy of SPM. Further clinical studies are needed to compare the antimicrobial efficacy of alcoholic and non-alcoholic herbal mouthwashes on oral and periodontal health. Nevertheless, on ethical grounds, it is imperative to respect the fact that culture, religion and customs may prohibit some individuals from using alcohol-based prescriptions. Although assessing the prevalence of oral *Candida* species was beyond the scope of the present investigation, we identified *C. parapsilosis* only in smokers. This result is in agreement with a study by Sampath et al,^39^ according to which *C. parapsilosis* is a yeast species present in the oral environment of tobacco-smokers. The possible role of oral yeast species in the initiation and progression of periodontal diseases in smokers warrants additional studies.

One limitation of the present study is that the participants were relatively young (mid-40s), and none of them had periodontitis. It is speculated that the anti-inflammatory and anti-fungal efficacy of SPM is compromised in comparison with CHX if used for the management of severe periodontitis. Moreover, radiographic examination was done only at baseline in the present study. This was primarily done to determine the extent of marginal bone loss (MBL) in the population and to identify patients with periodontitis. The present study had a relatively short follow-up; furthermore, it was ethically and scientifically challenging to justify exposing patients to ionising radiation after a short-term follow-up. It would have been interesting to include another group comprising tobacco smokers with periodontal inflammation. However, this requires additional studies with long-term follow-up. Furthermore, patients with a compromised immune system (such as patients with diabetes mellitus) were excluded. Raised glycemic levels (common manifestation in patients with poorly-controlled diabetes) is a risk factor for increased OCC.^19^ It is hypothesised that SPM are less effective than CHX in terms of reducing OCC among diabetic patients. This warrants additional well-designed and power-adjusted clinical trials.

## Conclusion

Both in cigarette smokers and non-smokers, CHX and SPM are effective in reducing periodontal soft-tissue inflammation after NSPT. Post-operative use of CHX is more effective than SPM in reducing OCC.
